# Innovative mRNA Vaccine Approaches in Targeting Atherosclerosis: A New Era in Cardiovascular Therapy

**DOI:** 10.7759/cureus.74141

**Published:** 2024-11-21

**Authors:** Rahul Kumar, Gowri Krishnaperumal, Chitra Vellapandian

**Affiliations:** 1 Pharmacy/Pharmacology, SRM College of Pharmacy, SRM Institute of Science and Technology (SRMIST), Chengalpattu, IND; 2 Pharmacology, SRM College of Pharmacy, SRM Institute of Science and Technology (SRMIST), Chengalpattu, IND

**Keywords:** atherosclerosis, cardiovascular disease, low-density lipoprotein, mrna, pcsk9

## Abstract

Atherosclerosis, a major cause of cardiovascular disease (CVD), involves plaque buildup in arteries driven by inflammation, endothelial dysfunction, and lipid metabolism disturbances. Current therapies aim to reduce cholesterol through statins and proprotein convertase subtilisin/kexin type 9 (PCSK9) inhibitors, prevent blood clots with antiplatelet drugs like aspirin, and control inflammation, alongside lifestyle modifications. However, these approaches often fall short due to patient non-compliance and residual risks. This review explores emerging mRNA vaccine strategies targeting the complex mechanisms of atherosclerosis. These vaccines could produce therapeutic proteins to modulate inflammation by encoding sequences that inhibit pro-inflammatory cytokines such as interleukin-1β (IL-1β), interleukin-6 (IL-6), and tumor necrosis factor-alpha (TNF-α), stabilizing plaques. Key targets include interleukin-10 (IL-10) for plaque stability, PCSK9 for cholesterol regulation, and vascular endothelial growth factor (VEGF) for endothelial repair. Addressing these unmet needs, mRNA-based approaches offer the potential for more effective and personalized treatments for atherosclerosis. However, challenges remain, including difficulty replicating human atherosclerosis in preclinical models, regulatory concerns about long-term safety, and ensuring accessibility in low-resource settings. In addition, large and diverse clinical trials are needed to confirm the efficacy of these vaccines in reducing cardiovascular events.

## Introduction and background

Atherosclerosis: an overview of the disease and global burdens

The progressive accumulation of plaque in the arteries is the hallmark of the chronic disease atherosclerosis, which can cause serious cardiovascular consequences such as peripheral artery disease, coronary artery disease, and stroke. Endothelial dysfunction, or damage to the artery's inner lining that permits low-density lipoprotein (LDL) cholesterol to build up inside the arterial walls, is usually the first sign of the condition. The proliferation of immune cells, the creation of fatty streaks, and ultimately the formation of atherosclerotic plaques, which have the potential to stiffen and constrict the arteries over time come next. Plaque rupture can happen in advanced stages, which can result in blood clots obstructing blood flow and frequently causing heart attacks or strokes.

Atherosclerosis continues to be the world's greatest cause of illness and death, greatly increasing the burden of cardiovascular disease (CVD). The World Health Organization (WHO) estimates that 17.9 million fatalities worldwide are attributed to CVDs, with atherosclerosis being a major factor in the majority of these cases [1]. The disease affects both developed and developing countries, yet because of restricted access to healthcare and preventive measures, some regions of shallow and middle-income countries experience greater fatality rates.

Atherosclerosis develops as a result of several important risk factors. Elevated LDL cholesterol levels in hyperlipidemia hasten plaque build-up in the arteries. Elevated blood pressure, often known as hypertension, stresses the arterial walls more, hastening the disease's advancement. Lifestyle variables that exacerbate endothelial dysfunction and inflammatory processes, such as smoking, dramatically increase the risk. Furthermore, genetic predispositions might increase an individual's risk of developing atherosclerosis, frequently due to hereditary diseases related to lipid metabolism. The growing global burden of atherosclerosis highlights the need for improved prevention and treatment techniques, which are underscored by the combination of these risk factors.

Current treatment landscape: limitations and unmet needs

Nowadays, the main treatments for atherosclerosis are blood clot prevention, inflammation control, and cholesterol reduction as a means of lowering cardiovascular risk. Statins are a common prescription medication used to decrease LDL cholesterol, which is a major cause of plaque formation in the arteries. Statins function by preventing the liver's production of cholesterol by blocking the enzyme HMG-CoA reductase. PCSK9 inhibitors, which are monoclonal antibodies that improve the liver's capacity to remove LDL cholesterol from the bloodstream by blocking the PCSK9 protein, increase the number of LDL receptors that are accessible to do so. This is another family of cholesterol-lowering medications. Furthermore, in individuals with advanced atherosclerosis, antiplatelet medications such as aspirin are used to prevent clot formation and lower the risk of heart attacks and strokes. The cornerstone of managing atherosclerosis consists of these treatments combined with lifestyle modifications [2].

The therapies that are now in use have considerable drawbacks notwithstanding their effectiveness. Residual cardiovascular risk is a significant concern, as patients continue to have cardiovascular events even after taking statins or other medications to lower their target cholesterol levels. This emphasizes how intricate and multifaceted atherosclerosis is, and how, in addition to cholesterol, endothelial dysfunction and inflammation also play significant roles. Drug resistance is another drawback; certain people only slightly lower their cholesterol or do not respond well to statins. Patient non-compliance is also a problem because these drugs frequently need to be taken for an extended period. Patients may stop their therapy because they feel that taking daily medicine is too difficult or expensive, especially for more recent treatments like PCSK9 inhibitors. Lastly, side effects include injection-site responses, muscle soreness, liver enzyme abnormalities with statins, and possible long-term effects with PCSK9 inhibitors can make patients reluctant to start medication or stop it altogether. These drawbacks highlight the unmet need for more potent and palatable treatments that target the root causes of atherosclerosis, such as persistent inflammation, and lower patients' residual cardiovascular risk.

Introduction to mRNA technology and its therapeutic potential

mRNA technology is a revolutionary method of therapeutic intervention, especially when it comes to using messenger RNA (mRNA) sequences to instruct cells to create particular proteins. The synthesis of a customized mRNA sequence that contains the genetic code needed to produce a target protein is the fundamental process of mRNA technology. After being given, the mRNA is carried into cells, usually with the help of lipid nanoparticles (LNPs), which act as messengers to shield the delicate mRNA from deterioration and promote its uptake into the cytoplasm. The encoded protein is created inside the cell by the ribosomes translating the mRNA, enabling the protein to perform its intended therapeutic function. Crucially, the effects of mRNA are transient and reversible due to its lack of genome integration, which lowers the possibility of long-term genetic alterations [3].

After being created initially for vaccinations, mRNA technology attracted a lot of attention after the COVID-19 vaccines were successful in teaching cells to make viral spike proteins, which in turn triggered an immune response. Nevertheless, mRNA technology has a wide range of potential uses outside of infectious diseases. By generating mRNA sequences that can lower cholesterol levels, alter inflammatory pathways, or produce protective proteins, researchers are investigating its potential application in the treatment of chronic diseases like as atherosclerosis. For example, mRNA-based treatments might be developed to express anti-inflammatory cytokines to lessen chronic inflammation, which promotes plaque development, or to block the synthesis of proteins like PCSK9, which controls cholesterol levels [4].

Due to its adaptability, mRNA technology is an attractive option for treating multifactorial, complicated diseases such as atherosclerosis, where targeting different pathogenic pathways may result in more individualized and successful approaches to therapy.

## Review

Pathophysiology of atherosclerosis: targets for mRNA-based vaccines

Lipid Metabolism and Plaque Formation

Dysregulated lipid metabolism, specifically LDL and their oxidized variants (oxLDL), is strongly associated with the pathophysiology of atherosclerosis and is a major factor in plaque production in artery walls. LDL normally transports cholesterol through the circulation so that cells can use it. Nevertheless, these particles can gather in the sub-endothelial region of arteries when there is an excess of LDL, particularly in the setting of hyperlipidemia. Here, LDL oxidizes to produce the highly atherogenic compound oxLDL. When OxLDL elicits an inflammatory response, immune cells like macrophages are drawn in and they consume the oxidized particles, transforming into foam cells. These foam cells get stuck inside the artery wall, which eventually leads to the formation of fibrous plaques and fatty streaks. Growing plaques cause the arteries to harden and constrict, reducing blood flow and raising the possibility that they could burst, resulting in cardiovascular events such as strokes and heart attacks [5].

mRNA-based vaccinations target certain proteins involved in cholesterol control, offering a novel way to regulate lipid metabolism and prevent plaque development. PCSK9 is a protein that inhibits the liver's capacity to eliminate LDL from the bloodstream by encouraging the deterioration of LDL receptors. It might be able to improve LDL receptor availability and dramatically lower circulating LDL levels by employing mRNA technology to suppress PCSK9 expression, which would lessen the risk of plaque formation. The main component of LDL particles, apolipoprotein B (ApoB), is another possible target. Because ApoB is necessary for the assembly and secretion of LDL, it is an excellent option for mRNA-based therapeutics that try to stop LDL synthesis before it starts. mRNA vaccines may provide a potent new method of preventing or delaying the development of atherosclerosis by targeting these important mediators of lipid metabolism. This strategy would be in addition to the current lipid-lowering medicines.

Inflammation in Atherosclerosis

Atherosclerotic plaques progress and become unstable due in large part to inflammation, which is a major factor in the formation and rupture of the plaques. When LDL builds up in the artery wall and becomes oxidized, an immunological response is triggered, which starts the inflammatory process of atherosclerosis. One of the first immune cells to be drawn to the site of plaque development is the macrophage. These cells become foam cells that make up the majority of the expanding plaque when they absorb oxidized LDL. As plaques develop, macrophages release pro-inflammatory cytokines such as tumor necrosis factor-alpha (TNF-α), interleukin-6 (IL-6), and interleukin-1β (IL-1β), which not only bolster local inflammation but also draw in T-cells and other immune cells. By the release of additional cytokines and chemokines, activated T-cells intensify inflammation even more, accelerating the formation of plaque in a feedback loop. The fibrous cap of the plaque becomes weakened over time by this ongoing inflammation, increasing the risk of rupture. A blood clot that forms as a result of a plaque rupture has the potential to cause potentially fatal cardiovascular events including strokes or heart attacks [6].

By modifying these inflammatory pathways, mRNA vaccines present a novel treatment strategy that may stabilize plaques and slow their growth. These vaccines can lessen the inflammatory response that causes plaque instability by encoding mRNA sequences that target pro-inflammatory cytokines or signaling molecules. For example, mRNA vaccines might be created to inhibit the synthesis of IL-1β or IL-6, two cytokines that are essential for both systemic and local inflammation in atherosclerosis. Lowering these cytokine levels may prevent inflammatory cells from being drawn to the plaque and delay its advancement. Moreover, TNF-α, a cytokine that contributes to plaque vulnerability and vascular inflammation, may be the target of mRNA vaccines. mRNA-based treatments can stabilize atherosclerotic plaques, limiting their rupture and lowering the total risk of cardiovascular events by attenuating the signaling pathways of these important inflammatory mediators. This method addresses the inflammatory aspect of atherosclerosis, which is still untreated in current therapy paradigms, and provides a viable way to supplement current anti-inflammatory medications.

Endothelial Dysfunction and Vascular Inflammation

One of the main causes of vascular inflammation and a crucial early step in the development of atherosclerosis is endothelial dysfunction. Vascular homeostasis is dependent on the endothelium, the thin layer of cells that lines blood vessels, which regulates blood flow, inhibits platelet aggregation, and manages inflammatory reactions. The endothelium's defense mechanisms are weakened when it sustains injury, which frequently results from things like smoking, high blood pressure, and excessive cholesterol. Because of this malfunction, the artery wall becomes more permeable, which permits LDL to enter and build up and start the atherosclerotic process. Furthermore, impaired endothelial cells create less nitric oxide (NO), a vasodilator that reduces vascular inflammation and enhances blood vessel relaxation. These alterations eventually increase oxidative stress, encourage the recruitment of immune cells, and aid in the development and advancement of atherosclerotic plaques.

Thus, one of the most important treatment objectives in the management and prevention of atherosclerosis is to target endothelial cells and restore their function. Because mRNA-based therapies encode proteins that can improve endothelium repair and lower oxidative stress, they have great potential in this field. vascular endothelial growth factor (VEGF), a protein that promotes endothelial cell repair and regeneration, is one possible target. Endothelial repair and vascular integrity may be supported by providing mRNA encoding VEGF or other endothelial growth factors. The enzyme nitric oxide synthase (NOS), which generates nitric oxide, is another significant target. Vasodilation and vascular inflammation may be improved and nitric oxide generation may be restored with the use of mRNA therapies that upregulate NOS expression. Furthermore, mRNA-based strategies may target antioxidant enzymes like glutathione peroxidase or superoxide dismutase (SOD) to reduce oxidative stress, which is a major cause of endothelium damage. mRNA treatments can improve endothelium repair, minimize oxidative damage, and reduce inflammation to restore vascular health and decrease the development of atherosclerosis [7].

mRNA vaccine design for atherosclerosis: mechanisms and molecular targets

Encoding Antigens to Regulate Lipoprotein Metabolism

mRNA vaccines are gaining traction as a cutting-edge method of treating atherosclerosis by focusing on important molecular pathways related to lipid metabolism, specifically those that control the levels of low-density lipoprotein cholesterol (LDL-C). PCSK9 is a protein that is essential for regulating cholesterol and is one of the most promising targets. By binding to liver cells' LDL receptors and designating them for destruction, PCSK9 lowers the liver's capacity to remove LDL cholesterol from the bloodstream. One of the main risk factors for atherosclerosis is elevated LDL-C levels because LDL builds up in artery walls and helps plaque develop. Targeting PCSK9, mRNA vaccines function by encoding mRNA sequences that either negate PCSK9's activity or prevent it from being produced. After the body receives the mRNA, cells start to make proteins that inhibit PCSK9. This keeps more LDL receptors active and greatly improves the body's ability to remove LDL from the blood. For individuals who are resistant or intolerant to current medications, early research has shown that these mRNA vaccines can effectively lower LDL-C levels, providing a novel, perhaps safer alternative to statins and monoclonal antibodies [8].

Novel mRNA targets beyond PCSK9 are being investigated to further control lipid absorption and enhance lipid clearance. Angiopoietin-like protein 3 (ANGPTL3) is one such target. It is a protein that inhibits lipoprotein lipase and is involved in the build-up of cholesterol and triglycerides in the bloodstream. By enhancing the activity of lipoprotein lipase, which breaks down triglycerides and facilitates their elimination from the bloodstream, mRNA vaccines that decrease ANGPTL3 may improve lipid clearance. ApoB, a crucial structural element of LDL particles, is another newly discovered target. ApoB levels could be lowered using mRNA vaccinations, which would prevent LDL particles from forming and thereby lessen the total lipid load. Targeting proteins involved in cholesterol absorption is also of interest; one such protein is Niemann-Pick C1-like 1 (NPC1L1), which is in charge of intestinal cholesterol uptake. mRNA vaccinations may further lower blood lipid levels by reducing dietary cholesterol absorption through inhibition of NPC1L1. By addressing several facets of lipid metabolism, these developments in mRNA technology open up new possibilities for the management of atherosclerosis and hold promise for extremely targeted and potent therapies.

Targeting Inflammatory Pathways With mRNA-Based Vaccines

One intriguing approach to tackling the chronic inflammation that propels the evolution of atherosclerosis is to target inflammatory pathways with mRNA-based vaccines. Atherosclerotic plaque formation and instability are largely attributed to inflammatory processes, where immune cells such as T-cells and macrophages release pro-inflammatory cytokines that prolong arterial inflammation. mRNA vaccines that encode anti-inflammatory proteins like transforming growth factor-beta (TGF-β) and Interleukin-10 (IL-10) can be created to combat inflammation.

Strong anti-inflammatory cytokine IL-10 reduces local inflammation within plaques by blocking the activity of macrophages and the synthesis of other inflammatory cytokines. TGF-β exhibits extensive anti-inflammatory properties as well, aiding in tissue regeneration and stifling the immune cells that fuel plaque development. mRNA vaccines, which encode these proteins, provide a novel approach to inhibiting the chronic inflammatory response linked to atherosclerosis by encouraging the body to create its anti-inflammatory medicines right at the site of inflammation.

mRNA technology has the potential to not only improve anti-inflammatory pathways but also inhibit pro-inflammatory cytokines that worsen atherosclerosis. IL-1β is a cytokine that is important in generating plaque instability and vascular inflammation, making it a prime target. Increased inflammation within atherosclerotic plaques is associated with elevated levels of IL-1β, increasing the likelihood of rupture. Immunizations based on mRNA can be engineered to either decrease IL-1β synthesis or counteract its effects by expressing proteins that obstruct its signaling pathways. This strategy may be able to reduce the hyperinflammatory response that increases the risk of cardiovascular events and plaque vulnerability. Analogous tactics may be employed for additional pro-inflammatory cytokines, like IL-6 and TNF-α, which are also implicated in intensifying the inflammatory cascade in atherosclerosis. mRNA vaccines provide a versatile and potent tool to modify the inflammatory landscape of atherosclerosis by encoding mRNA sequences that target these cytokines, addressing both the underlying cause and the subsequent consequences of long-term inflammation in this disease [9].

Modulating Immune Response to Stabilize Plaques

mRNA vaccines present a novel strategy for controlling the immune system in atherosclerosis, potentially stabilizing plaques and lowering the chance of rupture. One of the main causes of cardiovascular events, including heart attacks and strokes, is plaque instability, which is mostly brought on by immune system maladaptation in response to lipid accumulation in arterial walls and chronic inflammation. By teaching the immune system to identify and react to the fundamental causes of plaque formation more skillfully, mRNA vaccines can be developed to lower inflammation and increase plaque stability.

The capacity of mRNA vaccines to create proteins that promote immunological tolerance to antigens associated with atherosclerosis, such as oxLDL, is a crucial mechanism. The development of regulatory T-cells (Tregs), which are essential for inhibiting excessive immunological activation, may be aided by these vaccinations. mRNA vaccines may be able to reduce the chronic inflammatory response that fuels plaque formation and destabilization by increasing Tregs numbers. Furthermore, these vaccines may encode pro-inflammatory cytokines such as IL-10, which inhibit macrophage function and lower plaque-associated pro-inflammatory cytokine release. This may aid in preventing the development of foam cells and the build-up of necrotic core materials, two processes that compromise the plaques' structural integrity.

Another strategy makes use of mRNA vaccines to improve the removal of debris and dead cells from plaques using macrophage-mediated processes including efferocytosis. mRNA vaccines can decrease the size of the necrotic core and reinforce the fibrous cap that keeps the plaque from rupturing by increasing the effectiveness of macrophages in eliminating these apoptotic cells. Furthermore, since inflammatory chemicals like IL-1β are known to contribute to plaque instability, these vaccines can be made to specifically target and neutralize these molecules. The chronic inflammation that causes plaque rupture may be lessened by mRNA vaccinations by lowering levels of IL-1β and other pro-inflammatory cytokines.

All things considered, mRNA vaccines present a viable method of teaching the immune system to identify and control important variables linked to the advancement of atherosclerosis. These vaccines can stabilize plaques, lessen their susceptibility to rupture, and eventually minimize the risk of potentially fatal cardiovascular events using specific modulation of immune cells and inflammatory pathways [10].

Advancements in mRNA vaccine delivery for CVDs

Lipid Nanoparticles (LNPs) and Their Role in mRNA Vaccine Delivery

Because LNPs can preserve delicate mRNA molecules, maintain stability, and increase efficacy through targeted distribution, they have become the ideal delivery technology for mRNA vaccines. Since mRNA molecules are fragile by nature and easily broken down by the body's enzymes, it is difficult to distribute them efficiently in the absence of protection. LNPs protect the mRNA from deterioration and promote cell uptake by encasing it in lipid layers. The mRNA is released by the LNPs into the cytoplasm of the target cells, where it is translated into therapeutic proteins. Ensuring that the mRNA reaches the targeted cells intact and functionally is crucial for the success of mRNA vaccines, especially those being researched for cardiovascular disorders like atherosclerosis.

The capacity of LNPs to be tailored for targeted delivery is one of the main reasons it is preferred for mRNA vaccine administration. Scientists can target particular tissues or cells, such as those implicated in cardiovascular disorders, by altering the composition of LNPs. For instance, LNPs can be engineered to specifically target smooth muscle cells, macrophages, or endothelial cells found in atherosclerotic plaques in the context of atherosclerosis. By guaranteeing that the therapeutic mRNA reaches the affected parts of the cardiovascular system, limiting off-target effects, and reducing the required dose, precision targeting increases the efficiency of the vaccination.

The goal of recent developments in LNP technology has been to increase the effectiveness of its delivery to cardiovascular tissues and atherosclerotic plaques. Scientists are creating new surface alterations for LNPs, like adding targeting ligands that attach to receptors that are overexpressed on cells linked to plaque. These alterations improve the absorption of mRNA in atherosclerotic lesions by enhancing the LNPs' ability to focus on vascular regions that are damaged or inflammatory. Furthermore, efforts are being made to improve the lipid composition and size of LNPs to extend their bloodstream circulation period and promote their accumulation in cardiovascular tissues Scientists are trying to make sure that mRNA vaccines for cardiovascular disorders can be administered with more efficiency and specificity, optimizing their therapeutic potential, by improving the design of LNPs. These developments create new opportunities for mRNA-based therapeutics in cardiovascular medicine in addition to raising the probability of a successful course of treatment.

Developing Tissue-Specific Delivery Systems

An important advancement in the effectiveness of mRNA vaccines for cardiovascular illnesses, especially in targeting atherosclerotic lesions, is the development of tissue-specific delivery mechanisms. Making sure the therapeutic molecules reach the precise cells implicated in the progression of the disease, such as endothelial cells, macrophages, and arterial smooth muscle cells, while reducing off-target effects, is a major problem in the delivery of mRNA-based therapeutics. To get around this, scientists are creating cutting-edge techniques to route mRNA vaccines precisely to the desired cardiovascular system regions.

Endothelial cell targeting is one promising strategy, this is significant since endothelial dysfunction is a precursor to atherosclerosis. The mRNA can be delivered precisely to endothelial cells by engineering LNPs or other delivery vehicles with ligands that bind selectively to surface receptors overexpressed on these cells, such as vascular cell adhesion molecule-1 (VCAM-1). Once inside the endothelial cells, the mRNA can induce the synthesis of proteins that either lessen oxidative stress, restore endothelial function, or stop more inflammatory cells from being recruited to the plaque formation location [11].

Macrophages are another important target in atherosclerosis since they are essential to both the destabilization and accumulation of plaque. Delivery system innovations are centered around delivering drugs to specific macrophages by the engineering of lipopolysaccharides (LNPs) to express ligands or antibodies that bind to specific receptors on macrophages, like scavenger receptors. With the help of these customized delivery methods, anti-inflammatory cytokines such as IL-10 or proteins that aid in the removal of cholesterol and dead cells from plaques can be introduced. By reducing plaque size and inflammation, this tactic may stabilize the plaques and lower their risk of rupture.

Moreover, the administration of mRNA vaccines targets artery smooth muscle cells (SMCs), which are involved in the fibrous cap that keeps plaques from rupturing. Through the creation of delivery systems that specifically target SMCs, scientists hope to improve fibrous cap stabilization and repair while lowering the risk of acute cardiovascular events. The mRNA vaccines can be directed to SMCs by ligands or antibodies that identify certain markers on these cells, such as smooth muscle actin, facilitating tissue healing and cap reinforcement. 

Novel approaches are being explored to include ligands or antibodies into the delivery systems to specifically target atherosclerotic lesions using mRNA vaccines, in addition to targeting these cell types. On the surface of the LNPs, for instance, can be affixed nanobodies or peptides that identify chemicals that are only expressed in inflammatory or injured artery areas. This lessens systemic exposure and possible side effects while enabling more precise localization of the mRNA to the plaque accumulation locations, improving the therapeutic effect. The precision and efficacy of mRNA vaccines for the treatment of atherosclerosis are expected to be greatly enhanced by these advancements in tissue-specific delivery systems, providing a more focused strategy to battle cardiovascular disorders.

Overcoming Delivery Challenges in CVDs

Ensuring the efficacy of mRNA-based therapeutics for cardiovascular illnesses requires overcoming delivery obstacles. The cardiovascular system poses several particular challenges that need to be overcome, such as restricted vascular permeability, the possibility of immune clearance, and the possibility of off-target effects that could compromise the safety and effectiveness of mRNA vaccinations. To enhance the delivery of mRNA to cardiovascular tissues and guarantee that it stays effective once it reaches the target site, delivery strategy innovations seek to address these challenges.

A primary obstacle in the delivery of mRNA to cardiovascular tissues and atherosclerotic plaques is the restricted vascular permeability found in the plaque-forming regions. Thickened artery walls are often found in areas where atherosclerotic lesions occur, which makes it challenging for therapeutic agents- such as mRNA vaccines enter deeply into the tissues. To overcome this, scientists are trying to modify the mRNA delivery systems' nanoparticle designs, such as LNPs, to make them more capable of overcoming these physical hurdles. For instance, the absorption of mRNA into sick areas can be enhanced by smaller nanoparticles or those designed with surface changes that interact with certain endothelium receptors. Furthermore, improving the concentration of mRNA in areas of need may involve focusing on leaky vasculature in inflammatory or damaged artery regions.

Immune system recognition and clearance of the messenger RNA (mRNA) or its delivery vehicle, which prevents the mRNA from reaching its target, is another major obstacle. Given the increased immunological activity in the vascular environment associated with CVDs, this is particularly problematic. Researchers are looking into ways to get around this, like PEGylation (the process of connecting polyethylene glycol to nanoparticles), which can assist elude the immune system, extend the period that the drug is in circulation, and stop the liver or kidneys from clearing the drug too soon. These alterations can protect against both immunological attack and quick degradation of the mRNA, increasing the probability that the therapeutic mRNA will reach the targeted cardiovascular tissues.

Furthermore, reducing off-target effects is essential to guarantee that mRNA vaccines do not unintentionally impact other tissues or trigger undesirable immune reactions. This is especially crucial for cardiovascular illnesses since unintentional consequences could make pre-existing symptoms worse. Precision-targeting techniques, such as adding ligands or antibodies to the delivery vehicles that direct the mRNA precisely to the atherosclerosis-causing cells, such as macrophages or endothelial cells, are being developed to address this. By improving the targeting mechanism, these strategies lessen the possibility that non-target tissues will absorb the mRNA, strengthening the therapeutic focus and lowering the possibility of adverse consequences.

Enhancing the half-life and persistence of mRNA once it reaches cardiovascular tissues is the final significant obstacle. The therapeutic window for mRNA is limited due to its inherent instability and quick degradation by enzymes. Researchers are working to optimize the chemical structure of mRNA molecules, such as by changing their nucleotides or employing self-amplifying mRNA, which may multiply within cells to promote sustained protein production over an extended period, to increase the stability and durability of mRNA in the body. These advancements in mRNA design can guarantee that the therapeutic effect lasts long enough to have a significant effect on cardiovascular tissues, especially in terms of lowering inflammation and plaque development.

Through the resolution of these delivery issues, which include boosting mRNA half-life, decreasing immune clearance, decreasing off-target effects, and increasing vascular permeability, scientists are laying the groundwork for more potent and precise mRNA-based treatments for cardiovascular disorders, especially atherosclerosis [12].

Preclinical and clinical studies: assessing efficacy and safety

Preclinical Animal Models in Atherosclerosis Research

The effectiveness and safety of mRNA vaccines for the treatment of atherosclerosis are assessed in large part through the use of preclinical animal models. ApoE-deficient mice, which develop hyperlipidemia and atherosclerotic plaques resembling those observed in humans, are among the most commonly utilized models. These models offer important insights into the potential mitigation of plaque formation by mRNA vaccines that target important pathways in inflammation and lipid metabolism. Using these preclinical models, recent research has explored mRNA vaccines intended to influence inflammatory processes implicated in atherosclerosis and to suppress PCSK9, which modulates LDL-C levels [13].

Significant discoveries from these investigations have indicated encouraging outcomes in lowering LDL-C levels, a primary cause of plaque accumulation. Apart from decreasing cholesterol, mRNA vaccines that target inflammatory pathways have demonstrated notable decreases in markers of inflammation like IL-1β and TNF-α, both of which are essential for the advancement and instability of plaque. Moreover, preclinical studies have demonstrated that mRNA vaccinations might improve plaque stability, reducing the likelihood of plaque rupture-a major contributing factor to acute cardiovascular incidents such as strokes and heart attacks.

These results imply that mRNA vaccines may have a dual effect, reducing lipid levels and regulating inflammation to maintain pre-existing plaques. These preclinical models' performance opens the door for additional research and testing in human trials, raising the possibility that mRNA-based therapeutics will revolutionize the field by lowering the incidence of atherosclerosis and averting cardiovascular events [14].

Clinical Trial Data: mRNA Vaccines in Cardiovascular Therapy

Early-stage clinical trials exploring the potential of mRNA vaccines as a cardiovascular therapeutic have started to reveal how they may lower cholesterol and alter inflammation in atherosclerosis. These trials are investigating how mRNA vaccines can target important regulators of lipid metabolism, like PCSK9, and inflammatory pathways implicated in plaque development and destabilization. They are expanding on encouraging preclinical evidence. Although mRNA vaccinations have already transformed the treatment of infectious diseases (e.g., COVID-19), their use in CVDs is still in the early phases of clinical study.

The purpose of these trials is to assess mRNA vaccines for their potential to lower pro-inflammatory cytokines such as IL-1β and TNF-α, which are known to promote plaque progression, as well as LDL-C, a major driver of atherosclerosis. The priority is safety, and preliminary findings indicate that mRNA vaccinations are often well tolerated in individuals with CVD. Important safety information from these trials has shown that the side effects, which are comparable to those seen with mRNA vaccines for infectious diseases, are minor injection site responses, temporary flu-like symptoms, and weariness [15].

Scientists are keeping a careful eye on biomarkers to evaluate how well mRNA vaccinations lower the risk of CVD. These include well-known indicators such as LDL-C concentrations and high-sensitivity C-reactive protein (hs-CRP), an inflammatory marker that is frequently raised in heart conditions. These trials' preliminary results have indicated positive trends in cholesterol reduction and inflammatory regulation, but long-term data on plaque regression and cardiovascular event prevention are still lacking. As a novel therapeutic method for atherosclerosis, the early-phase data suggest that mRNA vaccines have substantial promise in addressing the unmet needs of lowering residual cardiovascular risk and plaque instability. However, to properly evaluate the safety and long-term advantages of these vaccinations in cardiovascular therapy, more research with bigger cohorts and longer follow-up times is required.

Translational Challenges: From Bench to Bedside

There are various obstacles in the way of transferring the preclinical model of mRNA vaccines for CVD to clinical application. Preclinical research in animal models such as ApoE-deficient mice has shown encouraging outcomes in lowering cholesterol, regulating inflammation, and stabilizing plaques, nevertheless, bringing these treatments to human populations requires filling in important gaps. The biochemical diversity of human atherosclerosis, which is impacted by several genetic, environmental, and lifestyle factors that are challenging to fully mimic in animal models, is one of the main obstacles. To guarantee that the treatments are safe and effective for a wide range of demographic groups, including individuals with various genetic backgrounds and underlying medical problems, it is crucial to test mRNA vaccines in diverse human populations [16].

Navigating the regulatory environment for mRNA vaccines in cardiovascular treatment is another significant challenge. The FDA and EMA, for example, have clear regulatory frameworks for assessing medications and biologics; but, given the innovative application of mRNA technology in the management of long-term conditions like atherosclerosis, safety procedures need to be reevaluated. Regulators will have to evaluate long-term safety risks very carefully, such as immune responses that aren't planned, effects that aren't on target, and the stability of the mRNA constructions over time. Furthermore, it will be critical to ascertain whether mRNA vaccines offer long-term benefits or if repeated doses are required to maintain cholesterol control and inflammatory modulation, as cardiovascular illnesses usually require chronic therapy.

Lastly, to evaluate the effectiveness of mRNA vaccines as well as their capacity to avert serious cardiovascular events like heart attacks and strokes, extended clinical trials are needed. To ensure broad application and assess if the early benefits seen in early-phase trials translate into clinical outcomes like reduced morbidity and death, these studies need to involve sizable and diverse populations. In addition, ensuring that mRNA vaccines for cardiovascular disorders can benefit all people globally-especially those in low-resource settings will require tackling health disparities in access to innovative therapeutics. To successfully transfer these cutting-edge treatments from the lab to the clinic, researchers, physicians, regulatory bodies, and businesses must work closely together to overcome these translational obstacles.

Combination approaches: enhancing the efficacy of mRNA-based therapies

mRNA Vaccines in Conjunction With Traditional Lipid-Lowering Agents

Using mRNA-based vaccinations in conjunction with conventional lipid-lowering medications like PCSK9 inhibitors and statins offers an intriguing way to improve the effectiveness of atherosclerosis therapy. While mRNA vaccines that target particular pathways, such as PCSK9, have the potential to lower LDL-C levels and modulate inflammation, their combination with proven treatments may have a synergistic effect that improves cardiovascular outcomes and leads to more thorough lipid control. The most popular medications to lower cholesterol are statins, which function by blocking HMG-CoA reductase, an enzyme essential to the manufacture of cholesterol, while PCSK9 inhibitors lower LDL-C by stopping the hepatic cells' LDL receptors from being degraded. By targeting different pathways, mRNA vaccines could be added to this therapeutic mix to treat residual cardiovascular risk that remains after statin and PCSK9 inhibitor therapy, as well as to provide an additional layer of lipid regulation.

Although many patients with atherosclerosis or high cardiovascular risk continue to have inferior outcomes even with existing medicines, clinical perspectives on this combo strategy are hopeful. An important breakthrough may be made possible by mRNA vaccines' capacity to target certain molecules involved in inflammation and lipid metabolism. This is especially true for individuals who are statin-intolerant or who have developed medication resistance. For instance, mRNA vaccines that encode PCSK9 inhibitors and lower LDL-C may boost the effects of already available PCSK9 inhibitors, lowering cholesterol and preventing plaque formation. Furthermore, because statins have some anti-inflammatory qualities, mRNA vaccines may be able to diminish vascular inflammation and stabilize atherosclerotic plaques via altering inflammatory pathways.

The significance of ongoing clinical trials investigating combination techniques is underscored by the promise of mRNA-based therapeutics as a supplement to conventional treatments. In addition to assessing the effectiveness of these combinations, these trials must assess the safety and tolerability of combining mRNA vaccines with PCSK9 inhibitors or statins. The ultimate objective is to create a multimodal therapy plan that targets the immunological, inflammatory, and lipid aspects of atherosclerosis, offering patients a more thorough and efficient means of lowering their risk of CVDs.

Combining mRNA Vaccines With Anti-Inflammatory Drugs

Anti-inflammatory medications and mRNA vaccinations together offer a potentially effective approach to the more thorough treatment of atherosclerosis. In addition to cholesterol buildup, persistent inflammation in the artery walls causes atherosclerosis by hastening the growth and instability of plaque. mRNA vaccines are being developed to target particular inflammatory pathways; however, by tackling different aspects of the disease process, combining them with already-available anti-inflammatory medications like colchicine or IL-1 inhibitors may offer further benefits. Colchicine, for example, has been demonstrated to lessen cardiovascular events by blocking inflammatory pathways, especially by reducing the generation of IL-1β and neutrophil activity, both of which are essential for plaque rupture and inflammation [17].

In addition, mRNA vaccines can combat the chronic inflammation linked to atherosclerosis by encoding anti-inflammatory proteins or molecules like TGF-β or IL-10 that directly control immune responses. Furthermore, through lowering inflammatory indicators and the risk of recurrent cardiovascular events, IL-1 inhibitors, such as canakinumab, which block the IL-1β signaling pathway, have shown cardiovascular benefits in clinical trials. These inhibitors may work in concert with mRNA vaccines that target inflammatory pathways to further reduce plaque burden and improve plaque stability.

This combo strategy is justified by the fact that mRNA vaccines provide precise targeting of particular molecules implicated in the inflammatory cascade, whilst anti-inflammatory medications such as colchicine or IL-1 inhibitors target broader inflammatory processes. When combined, they can lessen vascular inflammation in specific areas as well as general inflammation, offering a complete approach to stopping the advancement of atherosclerosis. Research on combination therapy in clinical settings may open up new avenues for preventing major adverse cardiovascular events (MACE), especially for patients who remain at high risk because of persistent inflammation even after receiving traditional treatments to decrease cholesterol. Furthermore, this strategy may be able to help those who still have residual cardiovascular risk even after managing their cholesterol to an ideal level, providing a more comprehensive paradigm for treating atherosclerosis [18].

mRNA Vaccines and Personalized Medicine

mRNA vaccines have great potential to advance customized treatment, especially when it comes to treating atherosclerosis. Precision cardiovascular treatment is made possible by mRNA technology's capacity to be customized to a person's specific genetic risk factors, lipid profiles, and inflammation indicators. As opposed to conventional treatments, which take a one-size-fits-all approach, mRNA vaccines allow for patient customization to target the unique molecular and genetic causes of atherosclerosis. mRNA vaccines that target lipid-regulating pathways, for example, maybe more beneficial for those with familial hypercholesterolemia or specific PCSK9 gene mutations than traditional medications in decreasing LDL-C levels.

Moreover, it is possible to tailor mRNA vaccines to each patient's specific inflammatory profile. Although the inflammatory pathways might vary from person to person, chronic inflammation is a major factor in atherosclerosis. mRNA vaccines could be customized to inhibit the particular inflammatory signals that are most active in a patient's illness process by evaluating the patient's cytokine levels (e.g., IL-1β, IL-6, TNF-α) and other inflammation markers. For example, a patient with increased markers of vascular inflammation may benefit especially from an mRNA vaccination intended to enhance the expression of anti-inflammatory proteins such as IL-10.

To determine who is most at risk for problems from atherosclerosis, genetic testing may be combined with personalized mRNA vaccinations. These vaccinations may help stop the development of new plaque and stabilize existing plaques by focusing on genetic polymorphisms or mutations linked to immunological responses or lipid metabolism, hence lowering the risk of cardiovascular events. The precision and effectiveness of the treatment are further improved by the capacity to customize the dose and delivery methods of mRNA vaccines according to each patient's unique profile.

Personalized treatment for atherosclerosis could benefit from the use of mRNA technology in order to maximize therapeutic benefits and reduce side effects. There is less chance of off-target effects or unneeded immune activation when only the routes pertinent to a patient's disease are targeted. This strategy represents a move toward precision healthcare, in which treatment plans are developed using a thorough understanding of a patient's biological composition, enabling more customized and successful control of atherosclerosis.

Safety considerations and potential risks of mRNA vaccines in atherosclerosis

Immunogenicity and Autoimmune Reactions

Concerns about immunogenicity and the possibility of autoimmune reactions must be taken into account while evaluating the use of mRNA vaccines for atherosclerosis. The induction of an immunological response is one of the main ways that mRNA vaccines work, which gives rise to worries regarding the possibility of immune system overactivation. The effectiveness of the vaccination depends on this immune activation, but excessive or unchecked immune responses may have unexpected consequences including systemic inflammation or the worsening of existing autoimmune disorders.

Since mRNA vaccines introduce synthetic mRNA sequences that encode certain proteins or antigens, there is a danger of autoimmunity. Theoretically, these antigens could unintentionally cause immune responses against the body's tissues even though their design targets pathways linked to atherosclerosis. This is especially likely if there is any molecular similarity between the vaccine-encoded proteins and endogenous proteins. Autoimmune illnesses could result from this, in which healthy cells are unintentionally attacked by the immune system. While there is a lack of widespread observation of these events in the current mRNA vaccine applications, like COVID-19, careful observation and long-term studies are required to assess this risk in the context of treating atherosclerosis, a disease that already includes immunological dysregulation [19].

The possibility that mRNA vaccines will cause inflammatory reactions outside of the targeted therapeutic range is another cause for concern. Since chronic inflammation within the artery walls is a hallmark of atherosclerosis, mRNA vaccines that target immune pathways need to carefully balance preventing the production of new inflammatory processes while also lowering detrimental inflammation. The destabilization of atherosclerotic plaques by overactivation of immune cells, including macrophages or T-cells, may increase the likelihood of plaque rupture and severe cardiovascular events, such as myocardial infarction or stroke.

Therefore, even though mRNA vaccines offer a promising new avenue for the treatment of atherosclerosis, extensive preclinical and clinical research is necessary to evaluate the safety profile of these treatments, especially regarding immunogenicity and autoimmune risk. Thorough monitoring of biomarkers, such as inflammatory cytokine levels (e.g., IL-1β, IL-6, TNF-α) and autoantibody presence, will be crucial in identifying premature indications of immune overactivation or autoimmune reactions. We can only be certain that mRNA vaccines for atherosclerosis are safe and efficacious for general usage in individuals with CVDs by conducting such thorough safety investigations.

Long-Term Safety of mRNA-Based Cardiovascular Therapies

As mRNA-based cardiovascular therapeutics approach clinical implementation, their long-term safety is an important factor to take into account. The effects of repeated doses over an extended period are a major issue, particularly since cardiovascular disorders like atherosclerosis may need continuous care to control the course of the disease. In contrast to vaccinations against infectious diseases, which are typically given once or twice, mRNA-based treatments for chronic illnesses might need to be given repeatedly or continuously to continue having a therapeutic effect. This calls into doubt the long-term toxicity and the cumulative effects of repeated dosage, especially about immune system activation [20].

The longevity of immune responses brought on by mRNA treatments is another crucial factor. Although it is ideal for these vaccines to trigger a strong and long-lasting immune response to lower cholesterol or stabilize atherosclerotic plaques, extended immune activation raises concerns that it may also cause autoimmune reactions or chronic inflammation. Sustained immune modulation has long-term impacts that need to be closely watched to prevent aggravating inflammatory pathways linked to CVDs. It may be possible to determine whether immune responses continue to be under control or if they start to hurt tissue damage or plaque destabilization by using biomarkers such as inflammatory cytokine levels and immune cell activation profiles.

The long-term presence of synthesized mRNA in the body carries possible hazards in addition to immune-related issues. Even though mRNA usually breaks down fast, its stability and duration of activity can be increased by using delivery technologies like as LNPs. On the other hand, extended mRNA persistence in cardiovascular tissues may result in unanticipated side effects, such as off-target effects when the mRNA affects non-cardiovascular tissues. To reduce these hazards, it will be essential to guarantee the specificity of mRNA delivery methods and minimize immune activation that is not targeted.

Furthermore, it is impossible to overestimate the possibility of long-term surveillance of patients undergoing mRNA-based cardiovascular therapy. Identifying any new safety issues entails routine evaluations of inflammatory indicators, lipid profiles, and general cardiovascular health. Long-term clinical trials and post-marketing surveillance will be required to fully comprehend the safety profile of these treatments, particularly in varied populations with a range of genetic origins and comorbidities. Ultimately, the effective application of mRNA-based therapeutics in the treatment of atherosclerosis and other cardiovascular illnesses will depend on resolving these long-term safety concerns through continued research and observation.

Mitigating Adverse Effects

Developing safe and efficacious mRNA-based vaccinations against cardiovascular illnesses, such as atherosclerosis, requires careful consideration of how to mitigate side effects. Dosage optimization is one of the most important tactics to lower adverse events linked to vaccinations. To minimize excessive immune activation or off-target consequences while eliciting an adequate therapeutic response, careful calibration of the mRNA dose is necessary. Lower dosages can still provide enough mRNA to produce the intended therapeutic effect while lowering the possibility of systemic adverse effects like inflammatory responses. The best dosage that maximizes effectiveness while reducing harm will be determined using dose escalation studies conducted during clinical trials.

Another key tactic to reduce negative effects is to improve delivery methods. While preventing accidental distribution to other organs, the targeted delivery of mRNA to particular tissues, such as endothelial cells or atherosclerotic plaques, can be improved by the use of advanced LNPs or other biodegradable delivery systems. The possibility of off-target effects, which occur when mRNA is supplied to healthy tissues where it is not required, can be greatly minimized by tissue-specific targeting. Further reducing adverse effects is the exact delivery of the therapeutic agent by the use of ligands, antibodies, or other targeting molecules that direct the mRNA to particular cell types, including macrophages or smooth muscle cells.

Reducing adverse effects also heavily depends on optimizing vaccination design. Developers can reduce the possibility of unwanted immune responses by carefully choosing and changing the mRNA sequences to encode only the pertinent proteins or antigens required for therapeutic impact. Furthermore, minimizing the possibility of extended exposure-which could trigger persistent immunological activation or other complications-can be achieved by making sure the mRNA constructs are designed to break down rapidly after serving their intended purpose. Furthermore, improvements in mRNA stability and delivery strategies will help to reduce the possibility of systemic or injection-site inflammation or immunological response.

Monitoring patient groups for early indications of negative effects is another tactic that enables action before major difficulties occur. Individuals who have experienced severe inflammatory disorders or autoimmune conditions in the past, for instance, may be more susceptible to vaccine-related side effects. Developers can customize medicines to reduce hazards by identifying these people and modifying the mRNA vaccination dose or delivery strategy accordingly.

Future directions and innovations in mRNA vaccines for CVDs

CVDs are among the intriguing new treatment areas where mRNA vaccines can be applied, thanks to their efficacy in the COVID-19 pandemic. The increasing body of research indicates that mRNA vaccines may be essential in the fight against atherosclerosis and maybe a broader spectrum of CVDs. This section delves into the potential applications of mRNA vaccines beyond the treatment of atherosclerosis, the progress made in mRNA platforms, and how personalized medicine and artificial intelligence (AI) can augment these developments.

Beyond Atherosclerosis: Expanding mRNA Vaccine Applications

Although the development of mRNA vaccines has mostly focused on atherosclerosis, this technique has several potential uses outside of inflammation and plaque formation. mRNA vaccines may provide therapeutic benefits for cardiovascular illnesses such as heart failure, hypertension, and vascular inflammation. For instance, by targeting the underlying molecular pathways of chronic illnesses like heart failure or high blood pressure, mRNA vaccines might be developed to encode proteins that control cardiac muscle activity or vascular tone.

Furthermore, there is a lot of potential for mRNA-based therapeutics to support myocardial regeneration and repair following a heart attack. Myocardial infarction causes scar tissue to grow and cardiomyocytes to be lost, which increases the risk of heart failure and causes long-term cardiac damage. mRNA vaccines have the potential to improve outcomes for patients who have suffered heart attacks by repairing damaged cardiac tissue through the encoding of proteins that stimulate angiogenesis or cardiomyocyte regeneration. This strategy may result in cutting-edge regenerative treatments that improve heart function at the level of cells in addition to managing symptoms.

Emerging Platforms for mRNA Vaccine Development

Not insignificant advances in mRNA technology are also influencing the future of mRNA vaccines for CVDs. Self-amplifying mRNA (siRNA), which has replication machinery that enables the mRNA to amplify itself inside cells, is one significant development. This potentially improves efficacy and eliminates the need for repeated injections by increasing protein production while needing fewer dosages of the vaccine. A platform like this would be especially helpful for long-term therapeutic benefits in chronic illnesses such as heart disease, where continuous protein expression is required.

A noteworthy advancement in RNA technology is circular RNA (circRNA), which is significantly more stable than linear mRNA due to the absence of open ends that can degrade. CircRNA is a great option for treatments that need to work consistently over an extended period, such as those that target inflammation or plaque formation in atherosclerosis. CircRNA may also result in longer-lasting protein production. A typical problem in the treatment of chronic cardiovascular illnesses, patient compliance might be improved and dosage frequency could be decreased as a result of this greater stability [21].

Apart from the RNA itself, new delivery platforms for the next generation are also being developed. By reducing off-target effects, advances in LNPs and other biodegradable polymers are making it easier to target particular tissues, such as heart muscle or vascular endothelium. These delivery methods can be adjusted so that mRNA vaccines get to the exact areas that need them most, such as the injured myocardium following a heart attack or the inflammatory vascular tissues in atherosclerosis.

Furthermore, the field of multi-target mRNA vaccines presents an intriguing prospect. Researchers can create vaccines that concurrently target many components of CVDs by encoding multiple mRNAs in a single formulation. A multi-target mRNA vaccination, for example, might promote endothelium repair and modulate inflammation in addition to reducing the metabolism of lipids (e.g., by encoding for PCSK9 inhibitors) to provide a holistic therapeutic approach against atherosclerosis.

Precision Medicine and AI in mRNA Vaccine Development

The application of AI and precision medicine to the development of mRNA vaccines is one of the most exciting new frontiers. AI is capable of analyzing enormous volumes of data, including lipid profiles, inflammatory markers, and genetic information, to pinpoint the risk variables associated with cardiovascular illnesses that are unique to each patient. Personalized mRNA vaccines that specifically target the molecular pathways causing a person's disease to progress can then be created using this information. An mRNA vaccine might be designed to target specific pathways, such as lipid metabolism or inflammatory responses if a patient has a genetic predisposition to high LDL-C levels or chronic inflammation.

AI can also predict patient reactions, enhance vaccination efficacy, and spot possible side effects early in the research phase by optimizing mRNA sequence design. This feature helps researchers to fine-tune their designs before the start of clinical trials, which ultimately speeds up the development process and expedites the translation of mRNA vaccines from bench to bedside.

By directing the selection of the most suitable delivery platforms and dose techniques based on individual patient profiles, precision medicine, and artificial intelligence can also improve the delivery of mRNA vaccines. Researchers can guarantee that the mRNA vaccine reaches its target area effectively, optimizing therapeutic advantages while limiting side effects, by customizing the delivery mechanism to each patient's specific demands [22].

## Conclusions

mRNA-based vaccines targeting inflammation, lipid metabolism, and plaque formation offer a transformative approach to treating atherosclerosis by focusing on molecular pathways like PCSK9. These vaccines promise tailored cardiovascular treatment, potentially improving patient compliance and reducing drug resistance.

However, challenges such as regulatory approval, safety concerns, and public acceptance must be addressed before clinical use in cardiovascular care. As mRNA technology advances, these vaccines could redefine CVD management by offering precise prevention and treatment, potentially reducing global rates of atherosclerosis and improving patient outcomes.
